# UV-B Radiation Stress Causes Alterations in Whole Cell Protein Profile and Expression of Certain Genes in the Rice Phyllospheric Bacterium *Enterobacter cloacae*

**DOI:** 10.3389/fmicb.2016.01440

**Published:** 2016-09-12

**Authors:** Jay Kumar, Piyoosh K. Babele, Divya Singh, Ashok Kumar

**Affiliations:** School of Biotechnology, Institute of Science, Banaras Hindu UniversityVaranasi, India

**Keywords:** UV-B radiation, phyllospheric bacteria, 2-D gel electrophoresis, proteome, gene expression

## Abstract

Among the different types of UV radiation, UV-B radiation (280-315 nm) has gained much attention mainly due to its increasing incidence on the Earth’s surface leading to imbalances in natural ecosystems. This study deals with the effects of UV-B radiation on the proteome and gene expression in a rice phyllospheric bacterium, *Enterobacter cloacae.* Of the five bacteria isolated from rice leaves, *E. cloacae* showed the highest level of resistance to UV-B and total killing occurred after 8 h of continuous exposure to UV-B. Reactive oxygen species were induced by UV-B exposure and increased with increasing duration of exposure. Protein profiling by SDS-PAGE and 2-dimensional gel electrophoresis (2-DE) revealed major changes in the number as well as expression of proteins. Analysis of 2-DE gel spots indicated up/down-regulation of several proteins under the stress of UV-B radiation. Thirteen differentially expressed proteins including two hypothetical proteins were identified by MALDI-TOF MS and assigned to eight functional categories. Both the hypothetical proteins (gi 779821175 and gi 503938301) were over-expressed after UV-B irradiation; gi 503938301 was characterized as a member of FMN reductase superfamily whereas gi 779821175 seems to be a structural protein as it did not show any functional domain. That the expression of certain proteins under UV-B stress is indeed up-regulated was confirmed by qRT-PCR. Transcript analysis of selected gene including genes of hypothetical proteins (*cp011650* and *cp002886*) showed over-expression under UV-B stress as compared to untreated control cultures. Although this study deals with a limited number of proteins, identification of differentially expressed proteins reported herein may prove useful in future studies especially for assessing their significance in the protection mechanism of bacteria against UV-B radiation stress.

## Introduction

Sunlight produces a broad spectrum of radiations from which the part reaching the Earth’s surface is composed of visible light, ultraviolet (UV-B and UV-A) and infrared radiation. Depletion of the stratospheric ozone layer due to anthropogenically released pollutants such as chlorofluorocarbons (CFCs), organobromides (OBs), and chlorocarbons (CCs) has resulted in an increase in UV-B radiation (280-315 nm) reaching the Earth’s surface (Crutzen, 1992). UV-B radiation is a cytotoxic wavelength range of solar radiation that exerts several structural and physiological effects on living organisms (Bancroft et al., 2007; Singh et al., 2013). Several biological effects of UV-B radiation include alteration in the structure of DNA, proteins, lipids, and other biologically important molecules, enhanced reactive oxygen species (ROS) generation, inhibition of such physiological processes as N_2_ fixation, photosynthesis and energy production in several organisms (Häder et al., 2007; Santos et al., 2012; Singh et al., 2013; Babele et al., 2015; Chudobova et al., 2015; Kurth et al., 2015). In particular, UV-B radiation present in the solar radiation directly damages DNA by inciting the formation of dimers in cellular DNA, including cyclobutane pyrimidine dimers and pyrimidine (6-4) pyrimidinone photoproducts (Rastogi et al., 2010a; Santos et al., 2012). Lesions formed in the DNA result in the blockage of DNA replication and RNA transcription leading to cell death (Santos et al., 2012; Chudobova et al., 2015).

The phyllosphere (plant leaf surface) represents a specialized microhabitat that supports growth of diverse microbes including bacteria, cyanobacteria, algae and fungi (Lindow and Brandl, 2003; Vorholt, 2012). As such the phyllosphere constitutes a harsh environment and only those microbes that are capable to cope with varied and fluctuating environmental stress conditions such as rapid changes in temperature, water, nutrient supply, osmotic stress and exposure to UV radiation (UVR) can colonize it and survive (Lindow and Brandl, 2003). Of the above environmental factors, solar UVR, particularly UV-B is perceived by the phyllosphere throughout the day and may damage the microbial flora, particularly bacteria (Vorholt, 2012). Ecological studies of the phyllosphere habitat clearly indicate that UV-radiation has a negative impact on individual microbial species and complex microbial communities at different trophic levels (Paul et al., 1997; Jacobs and Sundin, 2001; Kadivar and Stapleton, 2003; Gunasekera and Paul, 2006). Peanut plants exposed to solar UV-B radiation revealed major alterations in phyllosphere bacterial community composition as compared to control plants (Jacobs and Sundin, 2001). Similarly, the phyllosphere of maize plants exposed to UV-B radiation showed significant changes in bacterial community structure in comparison to untreated control plants (Kadivar and Stapleton, 2003). However, phyllospheric bacteria have developed a number of defense mechanisms to counteract the deleterious effects of UVR (Jacobs and Sundin, 2001; Jacobs et al., 2005). These include UVR avoidance, the production of carotenoid pigments and the expression of enzymes such as catalase and superoxide dismutase (Jacobs et al., 2005). Additionally, the role of mutagenic DNA repair determinant *rulAB* in conferring resistance to UVR has also been reported in the phyllospheric bacterium *Pseudomonas syringae* (Sundin and Murillo, 1999). However, as most previous studies were limited to a few phyllospheric bacteria, the need to conduct detailed investigations to understand and reveal the exact mechanism (s) of tolerance in bacteria found in UVR exposed harsh habitats such as leaf surface cannot be over-emphasized. Equally important would be the application of proteomics and genomics approaches in understanding the survival mechanism(s) of phyllospheric bacteria which form an integral component of all plants.

In our previous study, proteome analysis of the cyanobacterium, *Anabaena* L31 irradiated by artificial UV-B radiation showed significant changes in number as well as expression of various proteins (Babele et al., 2015). Certain novel UV-B responsive proteins were identified and their role in UV-B stress was implicated. To our knowledge, studies on the impacts of abiotic stresses have mostly been limited to terrestrial microbes and only meagre information is available for phyllospheric bacteria. Virtually, no attempt has been made to understand the mechanism of stress tolerance in phyllospheric bacteria by employing molecular approaches. Prompted by the above lacuna, we examined the effects of UV-B stress on bacteria isolated from rice phyllosphere. Our goals were to (a) study the changes in the proteome under UV-B stress, (b) identify selected UV-B responsive proteins, and (c) test the expression of selected genes by employing quantitative real time-polymerase chain reaction (qRT-PCR) to correlate the incidence of up- and/or down regulation of proteins. Hopefully, the findings of this study may provide new insights to understand how phyllospheric bacteria adapt to UV-B stress by modulating expression of UV-B responsive proteins and the relevant genes.

## Materials and Methods

### Isolation of Test Organism and Growth Conditions

Mature leaves of rice plants (Basmati rice variety HUBR 10-9) growing in full sunlight for the whole day were collected in the month of October, 2014 from the Agricultural farm of the Institute of Agricultural Sciences, Banaras Hindu University, Varanasi, Uttar Pradesh (25° 21′N, 83° 1′E and ~76 m above mean sea level) situated in the eastern gangetic plains of India. It is pertinent to mention that rice is mainly grown in rain fed areas during winter (Rabi) throughout India and the crops attain flowering stage during the month of October. For the isolation of bacteria, disease-free fresh leaves (0.5 g) were cut into small pieces and dipped in 10 mL sterile normal saline (0.9% NaCl) in a centrifuge tube. Tubes were vortexed vigorously to detach the associated epiphytic bacteria from the leaf surface. The resulting suspension was centrifuged at 2,000 × *g* for 5 min and the supernatant was collected in a separate tube. Different dilutions of the supernatant were made and 20 μL from each dilution plated on Luria–Bertani (LB) solid agar–agar medium. Plates were incubated at 37°C in a bacteriological incubator. Plates from each dilution were examined after 48 h of incubation and colonies showing distinct morphology in terms of shape, size, color, and texture were selected. Five colonies with distinct morphotypes were picked up and restreaked on fresh LB agar plates. Pure cultures of all the five putative isolates were obtained by repeated sub-culturing of a single colony on LB agar-agar medium. Growth of all the five isolates was also tested in liquid LB medium at 37°C in a shaker at 120 rpm (Orbitek LT, Scigenics Bioteck. Pvt. Ltd, India).

### UV-B Irradiation and Determination of Percentage Survival

The source of UV-B radiation was a UV-B lamp (Cat No. 3-4408, Fotodyne Inc., USA), emitting main output at 312.67 nm. UV-B intensity was measured by a Black-Ray Long Wave Ultraviolet Intensity Meter (UVP Inc., USA). The desired UV-B intensity was obtained by adjusting the distance between the UV lamp and test samples. Percentage survival of each of the isolates after UV-B radiation exposure was determined by colony count method. For determining percentage survival, twenty mL of actively growing liquid culture of each isolate was transferred in five Petri dishes (diameter 100 mm, with open lids) and exposed to UV-B radiation (2 Wm^-2^) for the desired time periods (1, 2, 3, 4, 6, and 8 h). Exposure of cultures to 2 Wm^-2^ of UV-B radiation was based on the fact that the intensity of UV-B in solar radiation averaged about 2.3 Wm^-2^ at global level (Madronich et al., 1998). Additionally, the low intensity of UV-B shows delayed effects and total killing of cyanobacteria/bacteria occurs after prolonged exposure, we had been performing experiments by using 2-2.5 Wm^-2^ of dose in all the experiments (Singh et al., 2013; Babele et al., 2015). Cultures were gently magnetically stirred to facilitate uniform exposure. Fifty microliter aliquots were withdrawn from each plate at known time intervals after UV-B treatment and spread on solid agar plates. Colonies appearing after 48 h of growth were counted in a colony counter and percentage survival was calculated. Unless otherwise stated, all the experiments were performed with log phase cultures having an initial dry weight of approximately 0.15 mg mL^-1^.

### Detection of Reactive Oxygen Species

Intracellular ROS production was detected by means of the probe 2′,7′-dichlorodihydrofluorescein diacetate (DCFH-DA) (Santos et al., 2012). Ten milliliter culture of the selected isolate was exposed to UV-B radiation (2 Wm^-2^) for different time periods up to 4 h. Irradiated and control cultures were harvested by centrifugation at 8,000 × *g* for 10 min at 4°C and washed with 10 mM potassium phosphate buffer. Pellets were suspended in 10 mL of 10 mM potassium phosphate buffer supplemented with 500 μM of DCFH-DA. Samples were incubated in the dark at 37°C for 1 h followed by brief sonication. One hundred μL of above samples from each set were taken out and protein content was determined by the Bradford method (Bradford, 1976). Fluorescence of the samples was measured with a spectrofluorometer (Varian, Inc., USA) with an excitation wavelength of 485 nm and emission wavelength of 519 nm. The fluorescence intensity at 519 nm was corrected against blank controls (cell extract without DCFH-DA) and thereafter normalized to protein content. ROS production is based on per mg protein.

### Protein Extraction

Whole cell protein was extracted as described previously (Kurian et al., 2006). Cells were harvested from unexposed (control) and UV-B exposed (treated) cultures (1, 2, 3, and 4 h exposure) by centrifugation at 10,000 × *g* for 10 min at 4°C in a Sigma Refrigerated Superspeed Centrifuge (Sigma, USA). The resulting pellets were suspended in 10 mM HEPES buffer (pH 7.2) supplemented with 1 mM phenylmethylsulfonyl fluoride (PMSF) and cells were broken by repeated freezing and thawing followed by mild sonication. The resulting suspension was centrifuged at 10,000 × *g* at 4°C for 10 min to remove cell debris. The supernatant obtained mainly contained cytoplasmic proteins. Chilled 10% (final concentration) trichloroacetic acid (TCA) in acetone was added to the supernatant and kept at -20°C for 2 h for the precipitation of proteins. Precipitate was collected by centrifugation at 14,000 × *g* at 4°C for 30 min. The pellet obtained was washed thrice with chilled acetone (95% v/v). Finally the sample was air dried and kept at -20°C till further use. If needed, salts and insoluble impurities, if any, were removed by a 2-D Clean-Up kit (GE Healthcare, UK). The protein content present in each sample was measured by Bradford method.

### SDS-PAGE Analysis of Protein

Forty microgram proteins of UV-B irradiated (1, 2, 3, and 4 h) and control cultures were loaded on a 12% SDS-PAGE and electrophoresed using SE 600 Ruby multiple gel-electrophoresis system (GE Healthcare, UK). Electrophoresis was performed at 50 mA for 3 h. Gel was stained with Coomassie Brilliant Blue R-250 (0.05% Coomassie Brilliant Blue R-250, 50% methanol and 10% acetic acid) for 12 h and destained with destaining solution (30% methanol and 10% acetic acid) for 24 h. The image was captured by means of the AlphaImager Gel Documentation unit (Alpha Innotech, USA).

### 2-Dimensional (2-D) Gel Electrophoresis and Spot Detection

2-Dimensional gel electrophoresis was performed with the proteins of control culture and 3 h UV-B exposed culture. The first dimension isoelectric focusing (IEF) was performed using Immobiline DryStrip (pH 4–7, 13 cm; GE Healthcare Bio-Sciences AB, Sweden). Protein sample (500 μg) dissolved in rehydration buffer (7 M urea, 2 M thiourea, 2% (w/v) CHAPS, 0.3% (w/v) DTT, and 0.5% (v/v) IPG buffer) was loaded onto each IGP strip and rehydrated for 14 h at 4°C in the dark. After rehydration, IEF was performed by the Ettan IPGphor 3 IEF system (GE Healthcare, UK) using the following steps; 2 h at 100 V, 2 h at 200 V, 1 h at 500 V, 1 h at 2000 V, 1 h at 3000 V, 1:30 h at 6000 V and 1:30 h at 8000 V until a total of 60000 Vh was attained. Equilibration of the IPG strips was done as described previously (Nouwens et al., 2002). The second dimension separation was carried out on 12% SDS-PAGE at 25 mA/gel using SE 600 Ruby multiple gel electrophoresis system (GE Healthcare, UK). Three gels were run for each protein sample of UV-B treated and untreated control cultures. Gels were stained with Colloidal Coomassie Blue G-250 (Candiano et al., 2004) and images were captured using the AlphaImager Gel Documentation unit (Alpha Innotech, USA).

The gels were scanned and protein spots analyzed by PDQuest^TM^ software version 8.0.1 (BioRad Laboratories, USA) for spot detection, quantification, background substraction, and spot matching between various gels. Spot quantification in the control and treated gels was performed by spot volumes (intensity × mm^2^) as described by Agrawal et al. (2014). The relative spot volume with respect to the UV-B treated and untreated control samples at desired time was compared by means of Student’s *t*-test. *P*-values less than 0.05 were considered statistically significant.

### Protein Spot Digestion and Mass Spectrometry

Differentially expressed proteins were identified by MALDI-TOF MS/MS (Matrix-assisted laser desorption/ionization-time of flight mass spectrometry). Selected protein spots were excised from the gel by manual picking using sterile one-touch spot picker and placed into separate microcentrifuge tubes. Trypsin digestion of the gel spots was done by the method of Shevchenko et al. (2006). MALDI-TOF MS analysis of protein spots was done at the Interdisciplinary School of Life Sciences, Banaras Hindu University, Varanasi. Data obtained were analyzed by Flex Analysis 3.3 and BioTools softwares (Bruker Corporation, Germany).

### Identification of Differentially Expressed Proteins

The proteins were identified by comparing peptide mass fingerprints at the NCBInr database using the Mascot search engine^1^. Search parameters allowed for oxidized methionines, carbamidomethylation of cysteines, and one missed cleavage site of trypsin with mass accuracy of ±30 ppm. Identification was based on the first ranking result and Mascot scores of >74 which indicated that the hits were significant.

### *In silico* Analysis of Hypothetical Proteins

UV-B responsive hypothetical proteins identified by MALDI-TOF MS analysis were further characterized by using bioinformatics tools. Protein BLAST (Basic Local Alignment Search Tool) was used to search similar protein sequences with known functions available at NCBI database. Top hits were selected and further analysis was performed using ClustalW to find the alignment of functional residues of proteins of known function with the sequence of hypothetical proteins (Larkin et al., 2007). Physico-chemical properties of proteins were determined using the ProtParam tool^2^ and database of *Enterobacter cloacae* subsp. *cloacae* strain 08XA1 as reference (Yan et al., 2012). InterProScan (version 5.0) server^3^ was used for the elucidation of protein family. Similarity search of both the hypothetical proteins was done by BLASTP. Phylogenetic analysis of the hypothetical proteins was carried out by using MEGA 6.06 software and tree was constructed by UPGMA (Unweighted Pair Group Method with Arithmetic Mean) method. Structure prediction of hypothetical proteins was done using EasyModeller version 4.0 *(*Kuntal et al., 2010). Three templates (PDBID-3SVL A.PDB- chromate reductase from *Escherichia coli*, 3S2Y A.PDB-chromate/uranium reductase from *Gluconacetobacter hansenii*, and 4H6P A.PDB-chromate reductase from *G. hansenii*) showing query cover above 93%, identity above 64%, and positives above 72% with respect to the hypothetical protein gi 503938301 were used for structure prediction. Validity of the structure was checked by the Ramachandran plot and quality of the predicted structure was checked by using PDBSum server.

### Quantitative Real Time-PCR (qRT-PCR) for Gene Expression

Five proteins, out of the 13 proteins identified by MALDI-TOF MS/MS, were selected for gene expression analysis by qRT-PCR for testing their regulation under UV-B stress. Among the selected genes, two were hypothetical protein genes (*cp011650* and *cp002886*) and three known protein genes namely, DNA gyrase inhibitor (s*bmC)*, peroxiredoxin (*ahpC*), and outer membrane protein (o*mpC*). *16S rRNA* was used as a bacterial reference gene (McMillan and Pereg, 2014). All the primer sets were designed by Primer 3 software and specificity was checked by Primer-BLAST (**Table 1**). Commercially synthesized primers (Integrated DNA Technologies Inc., USA) were used. Total RNA of the UV-B treated and control culture was isolated by means of RNA isolation kit (Ambion Life Technologies, USA) as per the manufacturer’s instructions. DNA was removed by DNase treatment. Complementary DNA was synthesized by employing cDNA synthesis kit (BioRad Laboratories, USA) according to manufacturer’s protocol. cDNA quantification was done by using Nanodrop ND-1000 spectrophotometer (Thermo Fisher Scientific, USA). qRT-PCR was performed on a MiniOpticon Real-Time PCR System (Bio-Rad Laboratories, USA) using SsoFast EvaGreen Supermix (Bio-Rad). Total reaction volume of 20 μL included: 10 nM of each forward and reverse primers, cDNA template (final conc. 20 ng) and the SsoFast EvaGreen Supermix (according to the manufacturer’s instructions). Each run included a non-template control (NTC). Real-time amplification for each gene including control and treated samples was performed in triplicate under identical PCR conditions. Thermal cycle was set as: 1 cycle at 95°C for 5 min, followed by 40 cycles at 95°C for 15 s, 53°C for 20 s, and 70°C for 30 s. The melting curve analysis was performed for all the assays; and the melting temperature of the product was determined using the melting curve program: 50–95°C, with a heating rate of 0.05°C per second and continuous fluorescence measurement. The Raw C_t_ values were extracted and analyzed using the CFX Manager Software (Bio-Rad, USA). The C_t_ values were automatically identified using the “Second Derivative Maximum Method” by the system software set with default parameters. The transcript expression level was normalized by the candidate reference gene (*16S rRNA*) relative to 0 h using the 2^-ΔΔCt^ method (Livak and Schmittgen, 2001). All the assays of qRT-PCR were performed under identical conditions for all the target genes.

**Table 1:** Primers used for amplification of different genes.

Genes	Primer sequence (5′->3′)	Amplicon size (bp)	Annealing temperature (*T*_m_)	Reference
*16S rRNA* (full)	8f: AGAGTTTGATYMTGGCTCAG	1500	52°C	Singh et al., 2009
	1495r: CTACGGCTACCTTGTTACGA			
*16S rRNA* (partial)	F: TATCCTTTGTTGCCAGCGGT	145	54°C	This study
	R: CGCTTCTCTTTGTATGCGCC			
*ahpC*	F: CCATACGCAGCATTTCGTCG	84	54°C	This study
	R: TGATCGACGCGAACGGCATC			
*sbmC*	F: TGAACAGCTGGTCATGTGGG	108	54°C	This study
	R: TCACAGCGCAGTTTTTCCGC			
*cp011650*	F: GAAGACCAGGATCTGACGCA	107	54°C	This study
	R: TCCCGGAGCAACCGCTGTCGG			
*cp002886*	F: TGTTCTGGGCGTTATGCTCT	136	54°C	This study
	R: TATCACTTCCAGCGGGTTCC			
*ompC*	F: TCTCTGACGACAAGAGTGCC	179	54°C	This study
	R: GAATTTCAGACCCGCGAACG			

*F, Forward; R, Reverse.*

### Statistical Analysis

All experiments were conducted in triplicate under identical conditions. Mean values and standard deviations were determined from three replicates of each treatment. A one-way *ANOVA* (analysis of variance) helped in confirming the significance of data according to Duncan’s multiple range test (DMRT) at *P* ≤ 0.05. OriginPro 8.5.0 (Origin Lab Corp., USA) software was used for the DMRT. Unless otherwise stated, values show the mean ± SD (*n* = 3).

## Results

### Identification of Bacteria and UV-B Radiation Sensitivity

**FIGURE 1:**
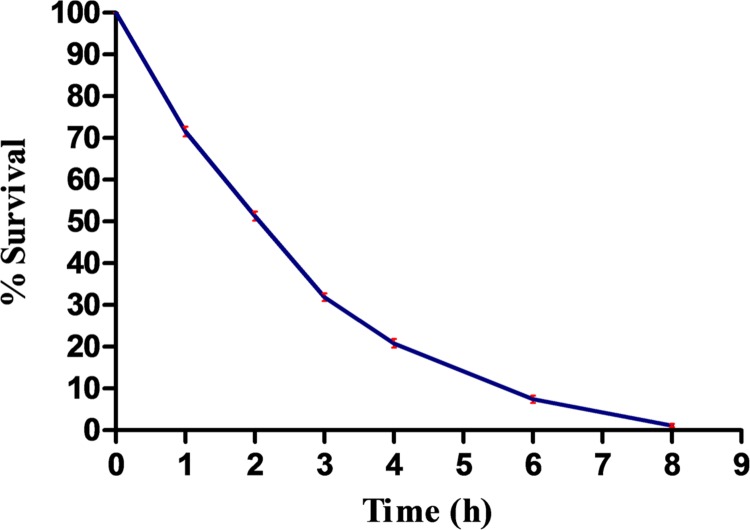
**Percent survival of *Enterobacter cloacae* following UV-B exposure.** An exponentially grown culture of *E. cloacae* was exposed to UV-B radiation (2 Wm^-2^) for desired time period and thereafter survival was determined. Results are based on the average of three experiments performed independently under identical conditions.

Five bacterial isolates (designated as RL-1 to RL-5) based on morphological characters and certain biochemical tests were isolated from the phyllosphere mature leaves of rice plant. Identification of all the five isolates was made by *16S rRNA* gene sequencing and accession numbers were obtained. These isolates were designated as: RL-1 (*Microbacterium sp.* SBT-6, KU376416), RL-2 (*Microbacterium sp.* SBT2, KU356922), RL-3 (*Enterobacter cloacae* RLI, KT161961), RL-4 (*Pantoea* sp. *SBT-4*, KU376415), and RL-5 (*Agrobacterium* sp. SBT-5, KU376420). Before assessing the impact of UV-B stress on protein profile, percentage survival of all the five isolates exposed to UV-B radiation (2 Wm^-2^ intensity) for varying time periods was determined to assess the tolerance level. Results showed decrease in percentage survival of all the isolates with increasing duration of UV-B exposure and complete killing of four isolates occurred at 6 h of continuous exposure to UV-B. However, one isolate namely, *E. cloacae* RLI (RL-3) showed complete killing after 8 h and retained ca. 52 % survival after 2 h of UV-B exposure (**Figure 1**). The observation that *E. cloacae* RLI shows higher level of tolerance to UV-B prompted us to select this isolate for all other experiments.

### Impact of UV-B Radiation Stress on ROS Generation

**FIGURE 2:**
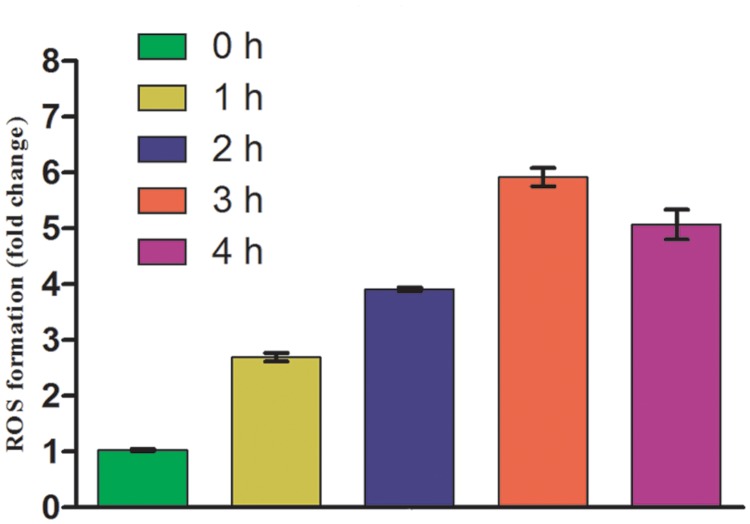
**Reactive oxygen species (ROS) generation after UV-B radiation treatment to *E. cloacae*.** Cultures were exposed to UV-B radiation (2 Wm^-2^) and ROS generation was measured at 0, 1, 2, 3, and 4 h as described in the Section “Materials and Methods”. ROS generation value is based on normalized protein content and expressed on the basis of per mg protein. Values on the y-axis represent fold change.

Once it became apparent that UV-B exposure causes loss of survival of *E. cloacae*, it was desirable to test the generation of ROS as its induction under oxidative stress has been well documented. As expected, ROS generation was enhanced in cultures exposed to UV-B radiation, and the level increased with increasing duration of exposure. The maximum increase was noted at 3 h as is evident from the increase in fluorescence of DCF (**Figure 2**). However, after 3 h of continuous exposure to UV-B, the fluorescence decreased possibly due to loss of cell viability or death.

### UV-B Stress-Induced Changes in Whole Cell Protein Profile

**FIGURE 3:**
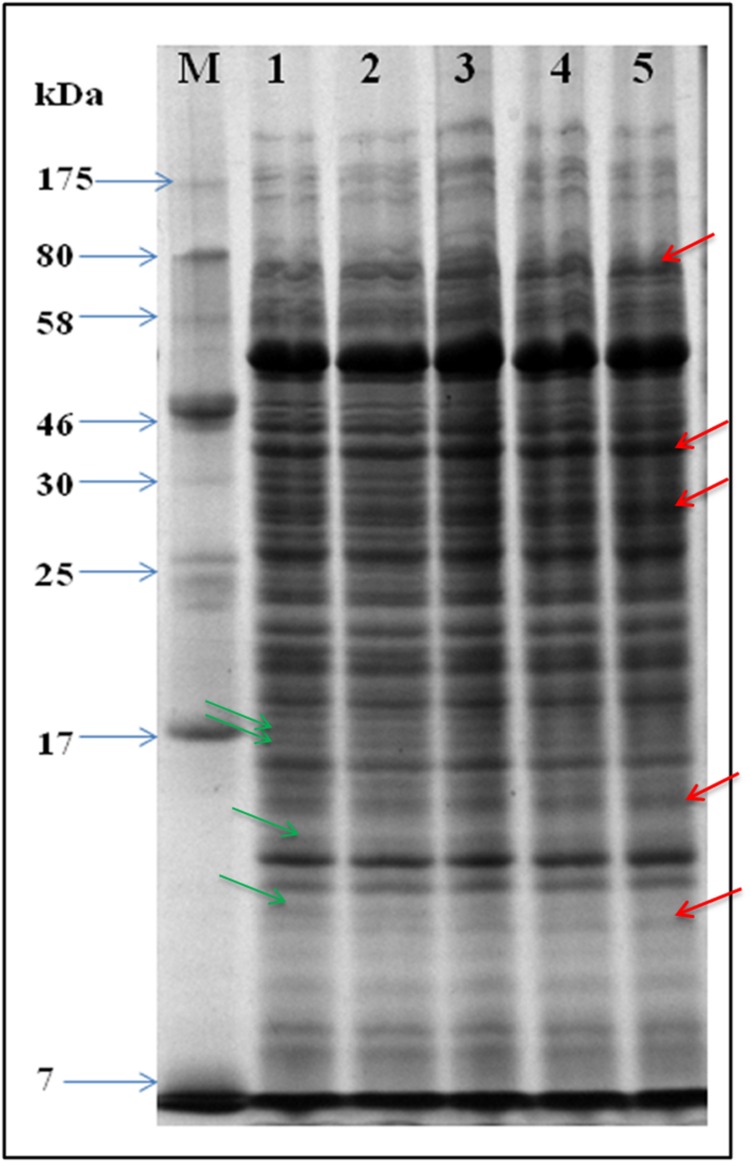
**SDS-PAGE gel showing whole cell protein profile.** Proteins were extracted from UV-B treated and control cultures and equal amounts of protein loaded in wells of 12% SDS-PAGE gel for separation. Lanes: M-molecular weight marker, 1- control, 2- 1 h, 3- 2 h. 4- 3 h, and 5- 4 h. Arrows in red and green color represent up-regulated and down-regulated proteins, respectively.

That, UV-B exposure causes loss of survival and induction of ROS generation as a result of oxidative stress aroused our interest to test alterations, if any, in the whole cell proteome of UV-B treated cultures. Accordingly, SDS-PAGE of proteins from the cultures exposed to UV-B radiation for varying time periods and control (unexposed) was performed. Analysis of gels based on electrophoretic pattern indicated alterations in the protein profile of UV-B treated cultures as compared to control cultures (**Figure 3**). Certain new protein bands appeared and a few were lost after exposure of cultures to UV-B radiation for 3 h. Additionally, the intensity of certain protein bands also decreased with increasing duration of exposure.

### Proteome Analysis

**FIGURE 4:**
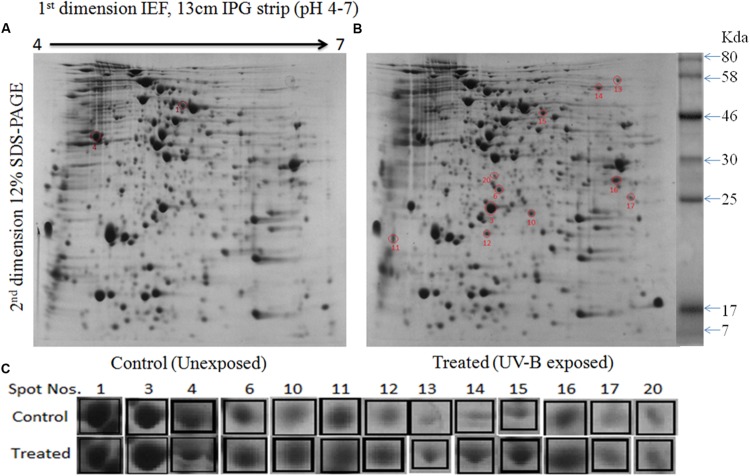
**2-Dimensional gel electrophoresis (2-DE) images of whole cell protein from *E. cloacae*. (A)** Control, and **(B)** irradiated with UV-B (2 Wm^-2^) for 3 h. Proteins were extracted and separated by 2-DE and visualized by CBB staining. Five hundred microgram proteins from each set were loaded on to pH 4–7 IPG dry strips for first dimension IEF followed by 12% linear vertical SDS-PAGE as the second dimension. **(C)** Expression pattern of selected proteins. Lane: C-control, and 3 h UV-B treatment. Numbers in bold represent the spot number as indicated on 2-DE gels. Proteins showing significant and reproducible changes were subjected to MALDI-TOF-MS. Master gel was prepared by PDQuest^TM^ software.

**FIGURE 5:**
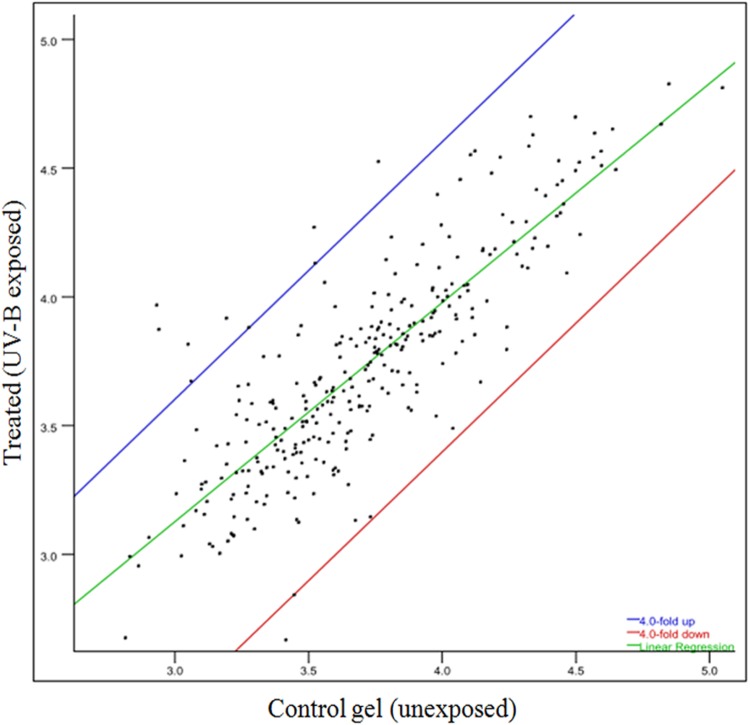
**Scatter plot showing level of expression of different spots of proteins.** Spots showing up- and/or -down regulation in 2-D gels are represented under different plots.

To gain a better insight of UV-B stress-induced changes in protein profile, 2-D gel electrophoresis was performed to find out changes in the proteome. It is evident from the analysis of gel (**Figures 4A,B**) that changes in both number and intensity of protein spots indeed occur upon exposure of cultures to UV-B. Analysis of gels showed 378 protein spots in untreated control and 384 spots in UV-B treated cultures of *E. cloacae*. Besides some changes in total number of spots, analysis of gels showed differential expression of several spots in UV-B treated cultures (**Figure 4C**). The data of scatter plot indicate that a number of protein spots show higher and/or lower level of expression in response to UV-B stress (**Figure 5**). Changes in expression level of proteins prompted us to focus our attention mainly on the protein spots which were showing either up- or down regulation on the basis of spot-to-spot comparison. Accordingly, thirteen protein spots showing more than twofold higher and/or lower level of expression from UV-B treated cultures as compared to untreated control (*p* < 0.05) were selected for MALDI-TOF MS analysis. Among the 13 identified proteins, 11 belonged to up-regulated group including two hypothetical proteins (**Figure 4C**) and 2 to down-regulated group. **Figure 4C** shows expression level of all the 13 identified proteins.

### Identification and Functional Classification of Selected UV-B Responsive Proteins

In functional studies, we compared the protein sequences with the secondary (or derived) protein databases that contain information on motifs, signature, and protein domains. Highly significant hits against different protein databases allowed us to derive the biochemical functions of query protein. **Table 2** summarizes the key findings related to bacterial processes responding to UV-B treatment based on the identification of representative proteins. All the 13 proteins identified in this study were sorted according to the functional categories defined by bacterial protein database. All the identified proteins were placed in eight different functional groups which included carbohydrate metabolism, energy metabolism, translation and transcription, fatty acid biosynthesis, nitrogen metabolism, antioxidative enzymes, membrane proteins and hypothetical (unknown) proteins (**Table 2**). There were two unknown proteins (hypothetical) namely, gi 779821175 and gi 503938301 among the 13 identified proteins.

**Table 2:** Differentially expressed proteins identified by MALDI-TOF MS in the cellular soluble fraction of *Enterobacter cloacae*.

Spot No.	Protein	NCBI accession No.	Gene	Length (amino acid)	Mass	Mascot score	Functional domain with InterProScan ID	Expression level
**A.**	**Carbohydrate metabolism**					
10	2,5-diketo-D-gluconic acid reductase	gi 654551989	*dkgA*	275	31028	175	IPR023210 NADP-dependent oxidoreductase domain	Up
16	D-ribose transporter subunit RbsB	gi 544826668	*rbsB*	296	30916	296	IPR028082 Periplasmic binding protein-like I	Up
**B.**	**Energy metabolism**					
14	ATP F_0_F_1_ synthase subunit alpha, partial	gi 746216253	*atpE*	490	52901	200	IPR000793 ATPase, F1/V1/A1 complex, alpha/beta subunit, C-terminal	Up
20	Glycerate dehydrogenase	gi 639772988	*hprA*	318	34842	83	IPR029752 D-isomer specific 2-hydroxyacid dehydrogenase	Up
**C.**	**Translation and transcription**					
11	DNA gyrase inhibitor	gi 495774886	*sbmC*	157	18326	102	IPR011256 Regulatory factor, effector binding domain	Up
**D.**	**Fatty acid biosynthesis**					
15	Beta-ketoacyl-[ACP] synthase	gi 654548503	*fabB*	405	42730	78	IPR014031 Beta-ketoacyl synthase, C-terminal	Up
**E.**	**Nitrogen metabolism**					
1	Aspartate ammonia-lyase	gi 504643254	*aspA*	478	52762	105	TIGR00839 aspA: aspartate ammonia-lyase	Down
**F.**	**Antioxidative enzymes**					
3	Peroxiredoxin	gi 503835101	*ahpC*	200	22316	203	IPR019479 Peroxiredoxin, C-terminal	Up
17	Chloroperoxidase	gi 544824839	*cpo*	278	30480	158	IPR029058 Alpha/Beta hydrolase fold	Up
**G.**	**Membrane proteins**					
4	Outer membrane protein	gi 697053451	*ompC*	370	40220	90	IPR013793 Porin, Gram-negative type,	Down
13	Periplasmic oligopeptide-binding protein	gi 556427175	*oppA*	543	61324	80	IPR030678 Peptide/nickel binding protein, MppA-type	Up
**H.**	**Unknown proteins**					
6	Hypothetical protein	gi 779821175	*cp011650*	312	35727	116	Phobius TRANSMEMBRANE Region of a membrane-bound protein predicted to be embedded in the membrane.	Up
12	Hypothetical protein	gi 503938301	*cp002886*	188	20373	176	IPR005025 NADPH-dependent FMN reductase-like	Up

### Prediction of Functional Domain and Phylogenetic Analysis of Hypothetical Proteins

Both the hypothetical proteins (gi 779821175 and gi 503938301) showed up-regulation under UV-B radiation stress. Till date, these two proteins have not been identified and characterized and their function is not known. The results of this study show that the protein gi 779821175 is encoded by a gene of *E. cloacae* located at locus AB285_13020 whereas gi 503938301 is encoded by a gene located at locus EcWSU1_04540. Domain analysis showed that gi 503938301 has a functional domain and shows similarity with FMN reductase superfamily. On the other hand, gi 779821175 has no functional domain and thus may be a structural protein. Physico-chemical properties of the protein gi 779821175 showed that it is rich in isoleucine (14.4%) and leucine (13.5%) whereas gi 503938301 is rich in leucine (10.1%) and glycine (9.6%). Details of functional domain analysis derived by InterProScan are presented in **Table 2**.

**FIGURE 6:**
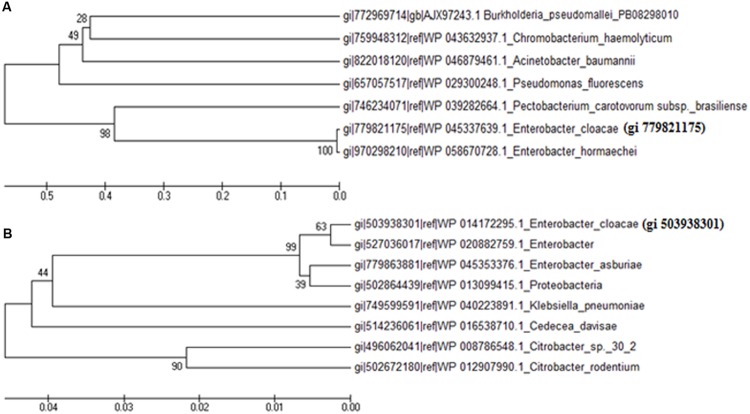
**Phylogenetic tree showing homology with proteins of other species of bacteria available in the database.** Phylogenetic tree of hypothetical proteins; **(A)** gi 779821175 and **(B)** gi 503938301.

From the BLAST results, it was noted that gi 779821175 does not show significant similarity with the related sequences of known functional proteins of any bacteria available in the NCBI database. It showed maximum identity (27%) with the putative membrane protein of *Burkholderia* sp. ABCPW 1. However, protein gi 503938301 showed 99% similarity with the FMN reductase enzyme of *Enterobacter*. With a view to revealing homology and phylogenetic relationship of gi 779821175 and gi 503938301 with the proteins of other species available in the NCBI database, a phylogenetic tree was constructed using reference strain *E. cloacae*. The phylogenetic tree indicates that the protein gi 779821175 shows 99% bootstrap value with the hypothetical protein of *Enterobacter hormaechei* (**Figure 6A**) but only 28% bootstrap value with the putative membrane protein of *Burkholderia pseudomallei* PB08298010. In the case of protein gi 503938301, it showed 99% bootstrap value with FMN reductase enzyme of *Enterobacter* and 90% bootstrap value with the hypothetical protein of *Citrobacter* sp. 30_2 (**Figure 6B**).

### 3-D Structure and Stereochemical Properties

**FIGURE 7:**
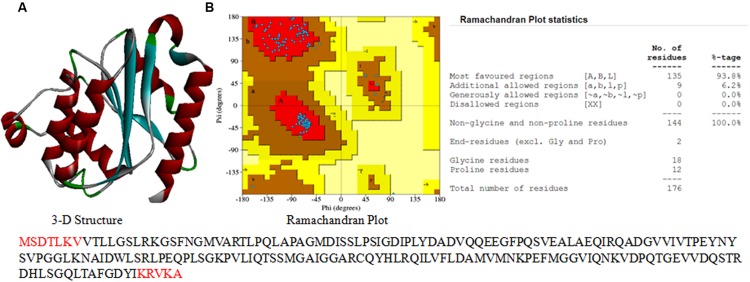
**3-D structure of hypothetical proteins. (A)** 3-D structure of gi 503938301, and **(B)** Ramachandran plot with statistics.

An attempt was made to construct 3-D model of both the hypothetical proteins using sequence similarity and available structures in the NCBI PDB database. A 3-D model of the gi 503938301 was constructed (**Figure 7A**) by using sequence identity and positive values with available templates in the NCBI PDB database. Additionally, validity of the structure was checked by the Ramachandran plot (**Figure 7B**). Analysis of plot generated for gi 503938301 revealed 93.8% residues in the most favored region, 6.2% in additional allowed region and no amino acid in disallowed region (**Figure 7B**). The values obtained are well within normal range for a high quality model. The best validated structure of gi 503938301 has been deposited in the PMDB database with PMDB-ID PM0080456. A 3-D model of the protein gi 779821175 could not be constructed as no suitable template was available in the PDB.

### Gene Expression Analysis

**FIGURE 8:**
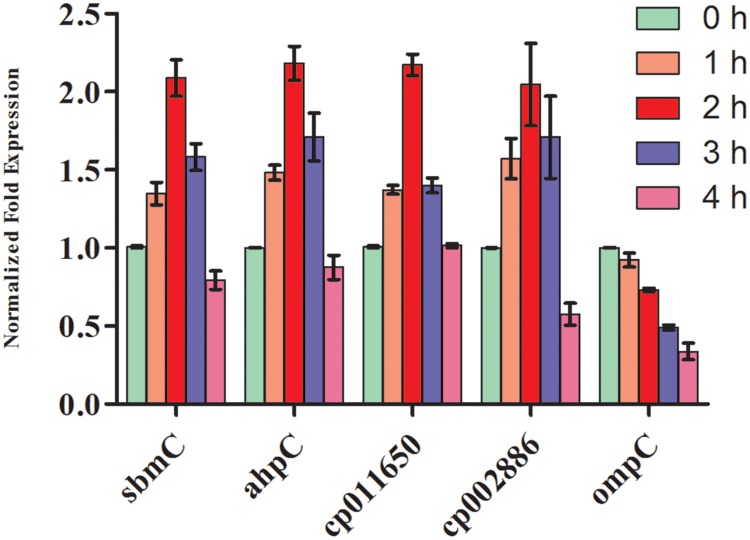
**Quantitative real time-polymerase chain reaction (qRT-PCR) for gene expression.** *E. cloacae c*ultures were exposed to UV-B radiation (2 Wm^-2^) for desired time period (1-4 h) and thereafter qRT-PCR was performed. Values represent normalized expression level of five selected genes both from control and UV-B exposed cultures.

To corroborate the findings of up-and/or down regulation of protein expression, transcript analysis of two hypothetical protein genes (*cp011650* and *cp002886*) and three known protein genes namely, DNA gyrase inhibitor (s*bmC*), peroxiredoxin gene (a*hpC*), and outer membrane protein (*ompC*) was made after exposure of cultures to UV-B radiation for varying time periods. qRT-PCR results revealed significant differences in the level of expression of target genes in the control and UV-B treated cultures. It is evident that C_t_ values of each target gene from cultures exposed to UV-B for varying time periods differ, suggesting differences in the level of expression (**Figure 8**). Of the five genes, *cp011650*, *cp002886*, s*bmC*, and *ahpC* showed more than twofold increase in expression at 2 h of UV-B exposure as compared to untreated control. In fact, the level of expression of the above four genes was higher even after 3 h of continuous exposure of cultures to UV-B, the maximum level being in the case of *ahpC* followed by *cp002886*. However, the level of expression decreased significantly after 4 h of exposure excepting *cp011650*. Notably, the expression of *OmpC*, a membrane protein gene showed gradual decrease with increasing duration of UV-B exposure, the level being more than half after 3 h of UV-B exposure in comparison to untreated culture (**Figure 8**). As expected, there was no change in the level of expression of reference gene (*16S rRNA*) irrespective of increase in time period.

## Discussion

Damaging effects of UV-B radiation on various metabolic processes of different organisms including bacteria and cyanobacteria are well documented (Häder et al., 2007; Rastogi et al., 2011; Santos et al., 2012; Singh et al., 2013; Chudobova et al., 2015; Oliveira et al., 2016). In the present study, it was noted that UV-B radiation exposure to five strains of phyllospheric bacteria resulted in the loss of survival and complete killing occurred after prolonged exposure. However, the effect on survival was differential: certain species such as *E. cloacae* elicited higher level of survival and total killing occurred after 8 h of continuous exposure of UV-B radiation. In general, phyllospheric bacteria show higher tolerance to UVR but sensitivity to UVR varies among different species (Jacobs and Sundin, 2001; Kadivar and Stapleton, 2003; Jacobs et al., 2005). The differences in tolerance to UVR could be due to the differences in defense mechanisms such as the presence of carotenoid pigment, DNA repair determinant *rulAB*, production of exopolysaccarides, synthesis of photoreactivating enzyme and the presence of novel UVR absorbing pigments (Jacobs et al., 2005; Saber et al., 2014). Such a conclusion is supported from the study conducted on the phyllospheric bacterium, *Pseudomonas syringae* wherein strains possessing *rulAB* maintained significantly larger populations than those lacking *rulAB* following UV-B irradiation (Sundin and Murillo, 1999). Similarly, contribution of pigmentation to field survival and population dynamics of *Clavibacter michiganensis* under UVR stress has been reported (Jacobs et al., 2005). Experiments conducted on two *C. michiganensis* pigment-deficient mutants under UVR stress showed significant loss of survival as well as reduced field population on peanut as compared to wild type strain (Jacobs et al., 2005). Findings of our study showing differential effects on survival of five phyllospheric bacteria are in agreement with the above reports. Enhanced level of tolerance to UV-B radiation exhibited by *E*. *cloacae* could be due to the presence of strong defense mechanism (s).

It has been well documented that the detrimental effects of UV-B radiation in most organisms are mediated by free radicals, which induce oxidative stress (Rastogi et al., 2010b; Singh et al., 2013; Das and Roychoudhury, 2014). It is also known that the over-production and accumulation of reduced oxygen intermediates including singlet oxygen can negatively affect proteins, DNA and lipids. Our results showed gradual increase in ROS level with increasing duration of UV-B exposure to the cultures of *E*. *cloacae*. Being consistent with the data of survival, this observation implicates the role of ROS in UV-B-mediated adverse effects on phyllospheric bacteria. However, reports on the role of induced ROS in biological and biochemical damage to bacteria are not only quite few but also mostly obtained indirectly from transcriptomic and proteomic studies based on the induction of antioxidative defenses upon exposure of bacteria to UV-B (Qiu et al., 2005; Matallana-Surget et al., 2009). Santos et al. (2013) reported that during UV-B exposure, single strand breakage and protein carbonylation occur and DNA damage is caused by different oxygen-free radicals in bacteria. However, more critical work is needed to understand the exact role of ROS formed during UV-B exposure on different metabolic processes of phyllospheric bacteria.

One major goal of our research was to study alterations in the proteome of the highly tolerant bacterium *E. cloacae* under UV-B stress. SDS-PAGE and 2-DE revealed significant changes in the whole cell proteome of *E. cloacae*. Alterations were noted both in the number and expression of proteins. A few new proteins were detected and certain proteins were lost. Alterations in quantity and quality of proteins are expected as it is an essential step of adaptive mechanism developed by any organism under UV-B stress (Babele et al., 2015). Accordingly, we selected thirteen protein spots showing more than twofold higher and/or lower level of expression from UV-B treated cultures and identified by MALDI-TOF MS. This was essential to better understand the metabolic events affected by UV-B stress and to test the possible role of these proteins in UV-B tolerance. It became evident from the results that these 13 identified proteins belonged to eight functional categories namely, carbohydrate metabolism, energy metabolism, translation and transcription, fatty acid biosynthesis, nitrogen metabolism, antioxidative enzymes, membrane proteins, and hypothetical proteins.

Among the up-regulated proteins of carbohydrate metabolism, 2,5-diketo-D-gluconic acid reductase and D-ribose transporter subunit RbsB are known to play important role in cell growth and energy storage. During UV-B stress, enhanced expression of these proteins is expected as cells require extra reductant and energy for survival. Similarly, energy generating enzyme ATP F_0_F_1_ synthase subunit alpha which is an energy metabolic protein plays key role in energy generation especially in ATP synthesis. Our results show that the transcript allowing ATP synthesis by F_0_F_1_ ATP synthase was up-regulated in the presence of UV-B stress. This enzyme is of great significance to the test organism as it uses a proton gradient to drive ATP synthesis and hydrolyzes ATP to build the proton gradient. Most probably, to overcome the UV-B stress, organisms require extra energy which might be compensated by the over-expression of ATP F_0_F_1_ synthase. DNA gyrase promotes supercoiling of DNA and regulates the replication of DNA and transcription of genes. Our research has showed increased expression level of DNA gyrase inhibitor during UV-B exposure, suggesting its role in the regulation of gene expression. Nakanishi et al. (2002) analyzed the interaction between DNA gyrase inhibitor and DNA gyrase of *Escherichia coli* and reported that gyrase inhibitor binds to the gyrase holoenzyme with higher affinity than to either the GyrA or GyrB subunit alone and thereby controls the supercoiling of DNA.

Beta-ketoacyl-[ACP] synthase (gi 654548503) is a fatty acid synthesis enzyme that catalyzes the condensation of malonyl-ACP with the growing fatty acid chain (Kauppinen et al., 1988). It has been documented that lipid peroxidation occurs during UV-B stress due to the generation of ROS leading to bacterial membrane damage (Gomes et al., 2013; Santos et al., 2013). To this effect, over-expression of beta-ketoacyl-[ACP] synthase might enable organisms to resynthesize the membrane under UVR stress and thus could mitigate the damaging effects of stress. Up-regulation of beta-ketoacyl-[ACP] synthase following UV-B stress in *E. cloacae* is in agreement with the above proposition and may play important role in maintaining the integrity of cell membrane.

One notable feature of the proteins involved in protection against oxidative stress is their expression and maintenance of homeostasis in stress condition. Henceforth, activation of antioxidative systems under stress may play crucial role in the protection mechanism of any organism. In this study, two antioxidative proteins, namely, peroxiredoxin (gi 503835101), and chloroperoxidase (gi 544824839) increased following UV-B stress and could possibly lower the damaging effects of increased ROS level in the cells. Similar to our observation, peroxiredoxin was reported to protect proteins from oxidative damage induced by ROS produced in the presence of abiotic stresses such as UV radiation, temperature etc. (Rhee and Woo, 2011). Peroxiredoxins have multiple roles including reduction of H_2_O_2_ and organic hydrogen peroxides and may act as molecular chaperones similar to heat shock proteins (HSPs) with roles in cell signaling. Similarly, chloroperoxidase functions as a catalase and catalyzes the dismutation of hydrogen peroxide in the presence of chlorine (Bengtson et al., 2013). Among the different types of chloroperoxidase, the heme-containing chloroperoxidase enzyme also shows peroxidase, catalase and cytochrome P450-like activities in addition to catalyzing halogenation reactions (Poulos et al., 1995). It has been reported that high ROS level induces the expression of genes encoding chloroperoxidase (Bengtson et al., 2013), a finding similar to our study as the UV-B stress caused increased production of ROS in *E. cloacae*. Altogether, features of chloroperoxidase point to its key role in the detoxification of ROS *vis-à-vis* preventing the killing of cells by toxic oxygen radicals. The level of periplasmic oligopeptide-binding protein, oppA (gi 697053451) also increased following UV-B exposure. This protein is a component of the oligopeptide permease, a binding protein-dependent transport system in Gram-negative bacteria; it binds peptides up to five amino acids long with high affinity (Richarme and Caldas, 1997). It has been reported that periplasmic substrate-binding protein promotes the functional folding of citrate synthase and alpha-glucosidase after urea denaturation and prevents the aggregation of citrate synthase under heat shock conditions. Interestingly, this enzyme forms stable complexes with several unfolded proteins, a function similar to that of a chaperone (Richarme and Caldas, 1997). Considering the above features, it appears that in addition to their main function in transport and chemotaxis, the role of bacterial periplasmic substrate-binding proteins might also be implicated in protein folding and protection against abiotic stresses. Equally important, the outer membrane protein, ompC (gi 697053451) showed decreased expression after UV-B irradiation to the cultures of *E. cloacae*. This finding is consistent with the report of the UV-C mediated alterations in protein profile of marine food borne pathogens, *Vibrio parahaemolyticus* and *V. alginolyticus* (Abdallah et al., 2012). It was reported that after UV-C irradiation several OMPs showed alterations including the appearance or disappearance of proteins and/or decrease in the expression level of certain OMPs. Most probably UVC stress proteins especially the down- regulated group of OMPs, protect bacteria against UV radiation similar to a report that the lack of OmpC and OmpF in the OMPs of *E. coli* and *Klebsiella pneumoniae* confers resistance to β-lactam antibiotics (Abdallah et al., 2012).

One important finding of our research relates to the identification and characterization of two proteins (gi 779821175 and gi 503938301) annotated as hypothetical proteins which are new and not reported so far from any bacteria subjected to UV-B radiation stress. *In silico* analysis revealed that gi 779821175 has 312 amino acid residues and contains a region of a membrane-bound protein predicted to be embedded in the membrane as per the analysis made by InterProScan server. However, it cannot be considered as membrane protein as it showed only 27% identity with the putative membrane protein of *Burkholderia* sp. ABCPW 1. Furthermore, as it could not be characterized in detail due to the lack of suitable template in the database, its function is also not known. Another protein, gi 503938301 which has 188 amino acid residues showed resemblance to the FMN reductase superfamily. As such this enzyme belongs to oxidoreductases class, and specifically acts on the CH-NH group of donors with FMNH_2_, NAD^+^ or NADP^+^ as acceptor (Liu et al., 1997). Notably, as enhanced expression of oxidoreductases is a well known event under abiotic stress, up-regulation of gi 503938301 could be useful for the organism in efficient electron transfer for different metabolic reactions under UV-B stress. Recently, the role of certain hypothetical proteins in the protection of UV-B stress has been reported in cyanobacteria (Babele et al., 2015). Further study is warranted to understand the exact function and role of gi 779821175 and gi 503938301 in mitigating the stress of UV-B radiation.

A few researchers have reported differential expression of various genes in different bacteria under UVR stress (Sundin and Murillo, 1999; Qiu et al., 2005). However, these studies were mostly based on the expression of a specific gene, and no attempt was made to correlate the expression level with respective proteins. In the present study, transcript analysis was performed targeting genes of up-regulated proteins. Results showed close correlation between genes showing up-regulated expression with respective up-regulated proteins under UV-B stress. Genes of four proteins showed more than twofold increased expression at 2 h of UV-B exposure suggesting their correlation with up-regulated protein expression. In summary, the data of proteome analysis and gene expression obtained in this study suggest that enhanced expression of certain proteins confers tolerance to *E. cloacae* against UV-B stress. However, further studies selecting several proteins from different phyllospheric bacteria are needed to unravel the complexity of the proteome following exposure of cultures to UV-B radiation stress.

## Conclusion

Several researchers have reported the occurrence and mode (s) of survival of a few phyllospheric bacteria from various plants but those studies were mostly conducted on the physiological aspects. Our study is the first of its kind and demonstrates changes in the proteome and gene expression of the phyllospheric bacterium *E. cloacae* isolated from the rice plant. Among 384 protein spots detected on 2-DE gels, 13 UV-B responsive proteins showing up or down regulation were identified by mass spectrometry. Among the identified proteins, 11 belonged to up-regulated and 2 to down-regulated group including two hypothetical proteins. Both the hypothetical proteins (gi 779821175 and gi 503938301) seem to be new as they have not been reported from any bacteria exposed to UV-B radiation stress. Notably, analysis of all the 11 up-regulated proteins suggests their role in UV-B stress tolerance. That UV-B stress does indeed promote enhanced expression of selected proteins is also evident from the transcript analysis of related genes. Among the five selected genes, four showed more than twofold increase in expression after 2 h of UV-B exposure to *E. cloacae*. It would be worthwhile to make detailed analysis of all the UV-B responsive proteins from a large number of phyllospheric bacteria so as to better understand the stress tolerance mechanism against UV-B as well as the roles of different classes of proteins in other vital metabolic processes, if any. Identification of differentially expressed proteins in this study may prove useful in future studies especially for assessing their significance in the adaptation mechanism of phyllospheric bacteria under UV-B radiation stress.

## Author Contributions

JK and DS conducted all the experiments and wrote the MS. PB did bioinformatics analysis and analyze all the data. AK helped in conducting experiments and writing the manuscript.

## Conflict of Interest Statement

The authors declare that the research was conducted in the absence of any commercial or financial relationships that could be construed as a potential conflict of interest.

## Footnotes

1 http://www.matrixscience.com

2 web.expasy.org/protparam/

3 http://www.ebi.ac.uk/Tools/pfa/iprscan/
